# Identification and validation of a novel overall survival prediction model for immune-related genes in bone metastases of prostate cancer

**DOI:** 10.18632/aging.204900

**Published:** 2023-07-25

**Authors:** Wen Bi, Weiming Guo, Gang Fan, Lei Xie, Changqing Jiang

**Affiliations:** 1 Department of Sports Medicine, Huazhong University of Science and Technology Union Shenzhen Hospital, The 6th Affiliated Hospital of Shenzhen University Health Science Center, Shenzhen, China; 2 Department of Urology, Huazhong University of Science and Technology Union Shenzhen Hospital, The 6th Affiliated Hospital of Shenzhen University Health Science Center, Shenzhen, China

**Keywords:** bone metastasis, prostate cancer, immune-related genes, tumor-infiltrating immune cells, MAVS

## Abstract

Immunotherapy has become a revolutionary treatment for cancer and brought new vitality to tumor immunity. Bone metastases are the most prevalent metastatic site for advanced prostate cancer (PCa). Therefore, finding new immunotherapy targets in PCa patients with bone metastasis is urgently needed. We conducted an elaborative bioinformatics study of immune-related genes (IRGs) and tumor-infiltrating immune cells (TIICs) in PCa bone metastases. Databases were integrated to obtain RNA-sequencing data and clinical prognostic information. Univariate and multivariate Cox regression analyses were conducted to construct an overall survival (OS) prediction model. GSE32269 was analyzed to acquire differentially expressed IRGs. The OS prediction model was established by employing six IRGs (MAVS, HSP90AA1, FCGR3A, CTSB, FCER1G, and CD4). The CIBERSORT algorithm was adopted to assess the proportion of TIICs in each group. Furthermore, Transwell, MTT, and wound healing assays were employed to determine the effect of MAVS on PCa cells. High-risk patients had worse OS compared to the low-risk patients in the training and validation cohorts. Meanwhile, clinically practical nomograms were generated using these identified IRGs to predict the 3- and 5-year survival rates of patients. The infiltration percentages of some TIICs were closely linked to the risk score of the OS prediction model. Some tumor-infiltrating immune cells were related to the OS. FCGR3A was closely correlated with some TIICs. *In vitro* experiments verified that up-regulation of MAVS suppressed the proliferation and metastatic abilities of PCa cells. Our work presented a thorough interpretation of TIICs and IRGs for illustrating and discovering new potential immune checkpoints in bone metastases of PCa.

## INTRODUCTION

Prostate cancer (PCa) is the most frequent cancer diagnosed in men; it accounts for 27% of diagnoses and ranks second in terms of fatalities in the United States [1]. It is anticipated that there will be 268490 new cases and 34500 deaths of PCa in 2022, based on the latest statistical data from the American Cancer Society [1]. Bone metastases are manifested in 70% of PCa patients in the advanced stage, and they were also present in 90% of individuals with metastatic PCa [2, 3]. Mechanisms that aggravate patients with PCa to develop bone metastases and immune regulation in bone metastatic PCa are not well understood, even though they contribute significantly to the mortality of men with advanced PCa [4].

Immunotherapy in cancer has made great progress, and tremendous immunotherapy clinical trials for various tumors have been witnessed in recent years. Tumor-infiltrating immune cells (TIICs) are closely associated with tumor progression and immunotherapy, as well as being biomarkers for prognosis and playing complex roles [5–7]. Chemokine CCL2 can recruit monocytes with highly expressed CCR2, while targeted inhibition of CCR2 can decrease the recruitment of M2 macrophages and induce tumor infiltration of activated CD8+ T cells [8]. Another chemokine, CXCL12, which can drive monocyte migration, could be induced by radiation therapy and trigger tumor-associated macrophage aggregation in tumor tissues [9]. IL-15 can up-regulate TIGIT and CD226 via tumor-infiltrating NK cells, increasing NK cell-mediated cytotoxicity and reducing tumor metastases [10]. Additionally, CD70 inhibits NK cell signaling, which is conducive to the immune regulation of B cell lymphoma and leukemia that express CD27 [11]. As stated, CCL2, CXCL12, IL-15, CD20, and CD70 are immune-related genes that have a certain significance for tumor development and immunotherapy. In parallel, PD-1 and PD-L1 have been the most successfully used immunotherapy targets, and antibodies targeting PD-1 and PD-L1 have exhibited promising efficacy in melanoma, lung carcinoma, and renal-cell cancer [12, 13]. Sipuleucel-T, however, is the most successful immunotherapy based on dendritic cells currently approved for advanced PCa [14, 15]. Therefore, finding new potential immune checkpoints in different tumors is of great significance. IRGs have been recognized as practical prognostic indicators and novel targets of various malignancies, including osteosarcoma [16], cervical cancer [17], colorectal cancer [18], and ovarian serous cystadenocarcinoma [19]. As a result, having a higher priority for knowledge of TIICs and IRGs will contribute to looking for particularly targeted molecules and may provide novel perspectives on PCa bone metastases.

For the current work, datasets on PCa bone metastases were obtained from GEO, TCGA, and cBioPortal databases. Differentially expressed IRGs and hub genes were confirmed from the GSE32269 dataset of the GEO database. Importantly, an IRG-based prognostic model was constructed and verified from the integrated data of TCGA-PRAD from the TCGA database and prad_su2c_2019 from the cBioPortal database. TIICs in primary and bone metastases of PCa and their correlation with risk scores were also analyzed and explored. Ultimately, our finding revealed that FCGR3A and MAVS might perform as appropriate immune targets for PCa bone metastases.

## MATERIALS AND METHODS

### Data preparation

Details on 2483 IRGs (Supplementary Table 3) were acquired from the ImmPort database (https://www.immport.org/resources). IRGs among differentially expressed genes (DEGs) in the GSE32269 dataset were filtered by the function “intersect” in the “dplyr” package. DEGs were authenticated from the GSE32269 dataset containing 29 metastatic bone marrow samples and four normal bone marrow samples using the “limma” packages of R software (version 4.2.1) using the criteria of an adjusted p-value < 0.05 and log2|fold change|>1. The volcano map was drawn using the “ggplots” package, and the heat map was plotted using the “pheatmap” package. Correlations between FCGR3A and PD-1, PD-L1, and CTLA4 were analyzed using the TIMER2.0 database (http://timer.comp-genomics.org/).

**Figure 1:**
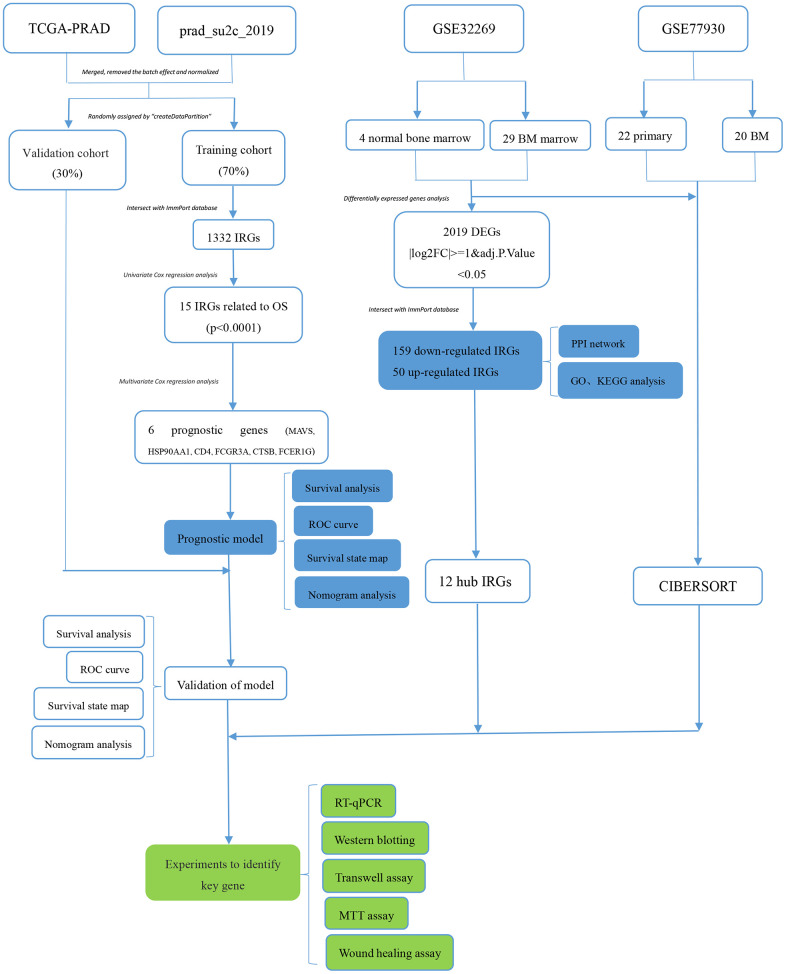
**The working flow chart of this study.** IRGs, immune-related genes; DEGs, differentially expressed genes; FC, fold change, BM, bone metastases; TCGA, The Cancer Genome Atlas; OS, overall survival; ROC, receiver operating characteristic; RT-qPCR, Real-time quantitative Polymerase Chain Reaction.

An appropriate dataset comprising RNA sequencing, FPKM values of 82 PCa bone metastases, and clinical survival information was downloaded from the cBioPortal database (http://www.cbioportal.org/study/summary?id=prad_su2c_2019) from Abida W’s study [20]. Considering the small number of patients with primary PCa in this dataset, the data from TCGA-PRAD was integrated. The TCGA-PRAD dataset, comprising a gene expression matrix, an annotation file, and clinical information for 505 PCa patients (1 metastatic and 504 primary tissues), was downloaded from UCSC Xena (https://xena.ucsc.edu/) [21]. Next, the same number of bone metastases and primary PCa samples were randomly selected from prad_su2c_2019 and TCGA-PRAD to integrate the new dataset. The new dataset consisted of an FPKM expression matrix and clinical information for 83 patients with bone metastasis and 83 patients with PCa *in situ*. Finally, the batch effect of the new dataset was removed via the “combat” function and normalized, then randomly divided into the training cohort (70%) and the validation cohort (30%) using the “createDataPartition” function in the “caret” package of R software (Figure 1).

### Function enrichment analysis

Gene ontology (GO) and Kyoto Encyclopedia of Genes and Genomes (KEGG) analyses of up- and down-regulated IRGs in GSE32269 were implemented to yield possible biological functions and signaling pathways using the R software “clusterProfiler” package. GO analyses consist of three parts: biological process (BP), cell composition (CC), and molecular function (MF).

### Identification of hub genes and biology networks

The STRING database (https://string-db.org/) is widely used for searching protein-protein interactions (PPIs), including the direct physical interaction between proteins and the indirect correlation between proteins. It is currently updated to version 11.5 and contains approximately 67.5 million proteins from over 14 thousand organisms and 200 billion interactions [22]. Fifty up-regulated and 159 down-regulated differentially expressed IRGs were imported into the STRING database and constructed into a visual network model by Cytoscape (version 3.9.1) [23]. In Cytoscape software for visualization, the cytoHubba plugin was used to calculate the betweenness and degree scores, which were important topological methods to evaluate the centrality of candidate genes [24]. The top 20 genes with the highest node scores were selected as candidate genes. Molecular Complex Detection (MCODE) is a graph clustering algorithm that can select key sub-modules and genes [25]. In the end, hub genes were mined based on the intersection of the results of betweenness, degree topological methods, and the MCODE algorithm.

### Building and verification of prognostic models

Details about preparing the training cohort and validation cohort data were provided above. Next, a univariate Cox regression analysis of IRGs was conducted in the training cohort via the “survival” package (version 3.4.0). The log-rank test was used for calculating the statistical significance of each IRG, and candidate genes were selected based on the standard of p<0.0001. Then, multivariate Cox regression was conducted to analyze the candidate genes and establish an optimal overall survival (OS) model. Finally, the risk score of each patient was calculated using the following formula: risk score = coef_gene1_ ×Exp _gene1_ + coef_gene2_ ×Exp_gene2_ +…+coef _gene(i)_×Exp _gene(i)_.

Patients were grouped based on the median risk score; those with higher risk scores were classified as high risk, while those with lower risk scores were classified as low risk. For evaluating the predictive power of the risk score on patients’ overall survival, Kaplan-Meier (K-M) survival curve analysis was implemented between two subgroups. The “timeROC” package (version 0.4) was used to map the time-dependent receiver operating characteristic (ROC) curve for evaluating the predictive ability of the above-mentioned prognosis model. Meanwhile, the same prognosis model was used to calculate the risk score and group in the validation cohort. Likewise, the survival and ROC curves were visualized using the above methods in the validation cohort. In order to make it easier for the OS prognosis model to be applied in the clinic, the regression modeling strategies (rms) package (version 6.3.0) was employed to build nomograms in the training and validation cohorts. Ultimately, the calibrate function in the “rms” package was used for mapping calibration plots to exhibit the error range of the prognostic model.

### Tumor-infiltrating immune cell analysis based on CIBERSORT

The CIBERSORT algorithm was implemented to calculate the proportions of TIICs in the GSE32269 and GSE77930 datasets, as well as training and validation cohorts. CIBERSORT is a widely used method for calculating and estimating the level of 22 TIIC components in tissues from their gene expression profiles [26]. The program reference document was provided as Supplementary Table 4.

### Cell culture and cell transfection

Four types of PCa cells were used in this study, including PC-3, DU-145, LNCaP, and 22Rv1, which were purchased from the Procell company (Wuhan, China). These four kinds of cells were cultured in RPMI-1640 (Procell, Wuhan, China) containing 10% fetal bovine serum (HyClone, USA) and 1% penicillin/streptomycin (Thermo Fisher, USA). Cells were all grown in an environment of 37° C and 5% CO_2_. Gene overexpression lentivirus MAVS mimics and their negative control were designed and constructed by Genechem (Shanghai Genechem Co., Ltd.). Cell transfection was carried out following the manufacturer’s instructions.

### Total RNA extraction and RT-qPCR

Following the instructions, total RNA was extracted from each cell and grouped with TRIzol reagent (Invitrogen, USA), then removed the gDNA and reversed transcribed into cDNA with PrimeScript™ RT reagent Kit with gDNA Eraser (Takara, Japan). Real-time quantitative PCR (RT-qPCR) was conducted using TB Green® Premix Ex Taq™ (Takara, Japan) according to the instructions. Primer sequences are exhibited in Supplementary Table 1.

### Capillary immunoblotting

For faster and more accurate detection of targeted proteins, we employed the Simple Western™ System (ProteinSimple, USA) for Western blotting. The cell lysis, protein extraction, and quantification methods utilized for each group after transfection were in line with those used in our former research [27]. Then, boiled protein samples, primary antibodies of MAVS (1:1000, Abcam), β-actin (1:5000, Affinity), Akt (1:1000, Abcam), and Capase-3 (1:5000, Abcam), and the Wes anti-rabbit detection module based on a published manuscript [28], were added to each well of the Wes Separation 12-230 kDa Capillary Cartridges. All Wes reagents were purchased from ProteinSimple, and the experiment was implemented strictly in compliance with instructions. Eventually, Image J software (version 2.9.0) was adopted for calculating the grey values of the images.

### Transwell assay

The Transwell assay is a method for simulating the migration and invasion processes of tumor cells *in vitro* by putting higher concentration serum on one side of the gel and cells on the other. Migration and invasion abilities are evaluated by counting the number of cells that traversed the 8-μm pore [29]. As previously illustrated [27], Transwell chambers (Corning, USA) with or without Matrigel (Corning, USA) were adopted for the invasion or migration assay. Finally, cells traversed from the pore were stained with modified Giemsa solution (Beyotime, China) and photographed under three random fields.

### Wound healing assay

The wound healing assay provides a cheap, simple, and convenient way to implement cell migration ability *in vitro* [30]. Linear scratches were made on each group of cells with a 200 μl sterilized pipette tip, and photographs were taken by microscope (Leica Microsystems GmbH, Germany) at the same location of scratches 0 h and 48 h later. Three cell scratch sites were randomly selected in each group, and the scratch areas were calculated by Image J software (version 2.9.0, Java 1.8.0_322).

### MTT cell proliferation assay

PC-3 and DU-145 cells were digested and counted after modeling successfully. Then the cells were seeded into a 96-well plate (2×10^3^ cells/well) and cultured at 37° C in an atmosphere of 5% CO_2_. Finally, the absorbance value was determined at 0 h, 24 h, 48 h, and 72 h using the MTT assay kit (Beyotime, China) following the instructions.

### Statistical analysis

The majority of statistical bioinformatics work was executed via R statistical software (version 4.2.1), comprising processing and normalization of bulk RNA sequence, DEG analysis, GO and KEGG enrichment analysis, CIBERSORT, survival analysis, ROC analysis, as well as Spearman correlation analysis. For univariate and multivariate Cox regression analysis, the function “coxph” in the “survival” package (version 3.4.0) was adopted.

The data for the *in vitro* validation experiment were exhibited as the mean ± standard deviation of three independent experiments. The GraphPad Prism software (version 8.0.2 for Windows) was deployed to conduct an unpaired student’s t test or one-way ANOVA to determine the differences between two or more groups and draw the statistical plots. Each experiment was repeated in triplicate for each sample. It was considered significant in statistics when the p-value was less than 0.05.

## RESULTS

### Differentially expressed IRGs in bone metastasis of PCa

**Figure 2:**
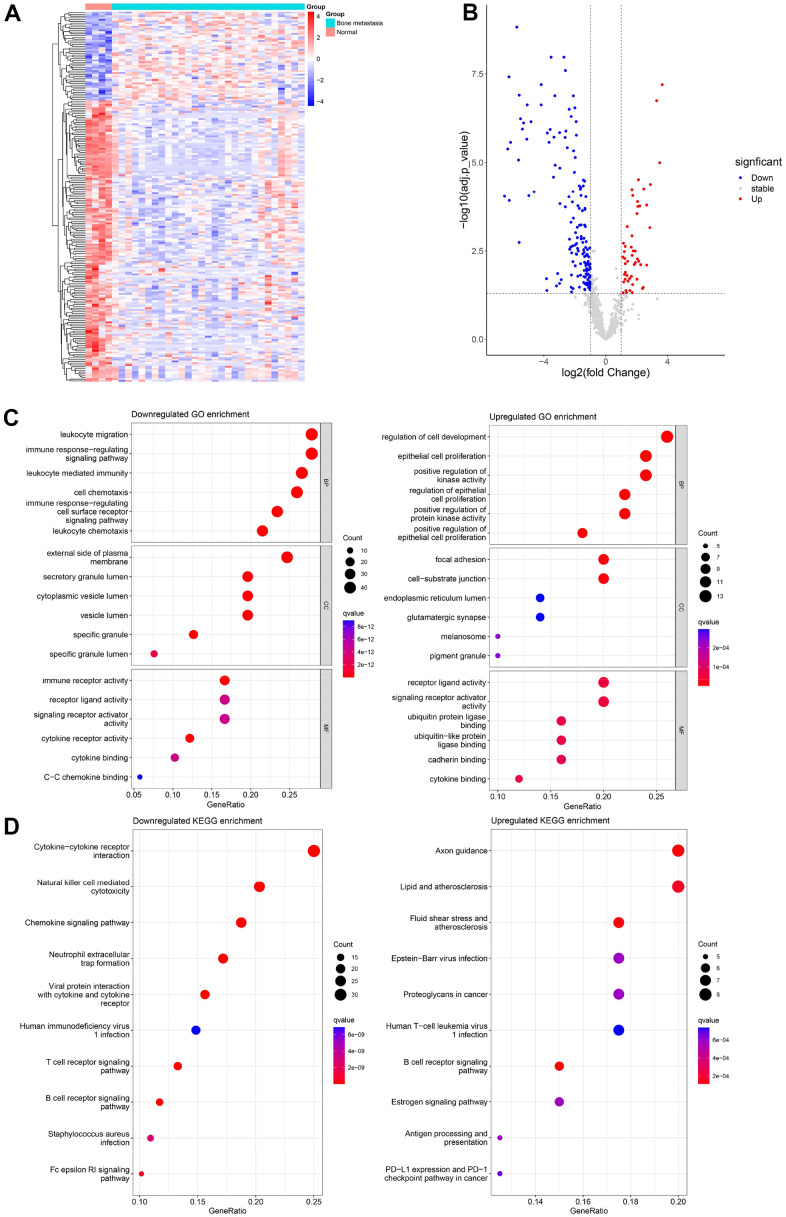
**Differentially expressed IRGs and their functional enrichment analyses.** Heat map (**A**) and volcano map (**B**) for differentially expressed IRGs in 4 normal bone marrow samples and 29 bone metastases of prostate cancer samples from GSE32269. GO (**C**) and KEGG (**D**) enrichment analysis for 159 down-regulated IRGs and 50 up-regulated IRGs.

GSE32269 from the GEO database was selected for DEGs analysis, containing 29 cases of PCa bone metastatic marrow and four normal bone marrow cases. A heat map of differentially expressed IRGs relative expression was exhibited in Figure 2A. Eventually, there were 209 IRGs that were differentially expressed; 159 of them were down-regulated, and 50 of them were up-regulated (Figure 2B and Supplementary Table 2).

### GO and KEGG pathway enrichment analysis

To better investigate the role of the above differentially expressed IRGs and potential mechanisms in the metastasis of PCa, GO enrichment analysis and KEGG functional enrichment analysis were conducted on those down- or up-regulated IRGs. The top six GO-enriched down- and up-regulated IRGs for each part are exhibited in Figure 2C. As for BP, 159 down-regulated IRGs were mainly involved in leukocyte migration, the immune response-regulating signaling pathway, leukocyte-mediated immunity, cell chemotaxis, the immune response-regulating cell surface receptor signaling pathway, and leukocyte chemotaxis. Fifty up-regulated IRGs were enriched in the regulation of cell development, epithelial cell proliferation, positive regulation of kinase activity, regulation of epithelial cell proliferation, and positive regulation of protein kinase activity and epithelial cell proliferation. Regarding the CC, down-regulated IRGs primarily constituted the external side of the plasma membrane, the secretory granule, the cytoplasmic vesicle lumen, the vesicle lumen, the specific granule, and the specific granule lumen. The up-regulated IRGs main components were focal adhesion, cell-substrate junction, endoplasmic reticulum lumen, glutamatergic synapse, melanosome, and pigment granule. A difference in CC indicates a different MF. Down-regulated IRGs influence immune receptor activity, receptor ligand activity, signaling receptor activator activity, cytokine receptor activity, cytokine binding, and C-C chemokine binding, whereas up-regulated IRGs influence receptor ligand activity, signaling receptor activator activity, ubiquitin protein ligase binding, ubiquitin-like protein ligase binding, cadherin and cytokine binding. Furthermore, KEGG pathway enrichment analysis revealed various pathways that IRGs enriched between up- and down-regulation (Figure 2D). The down-regulated IRGs are enriched in cytokine-cytokine receptor interaction, natural killer cell-mediated cytotoxicity, the chemokine signaling pathway, neutrophil extracellular trap formation, and viral protein interaction with cytokine and cytokine receptor. As for up-regulated IRGs, are enriched in proteoglycans in cancer, the B cell receptor signaling pathway, antigen processing and presentation, PD-L1 expression, and the PD-L1 checkpoint pathway (Figure 2D).

### Establishment of biological network and identified hub genes

**Figure 3:**
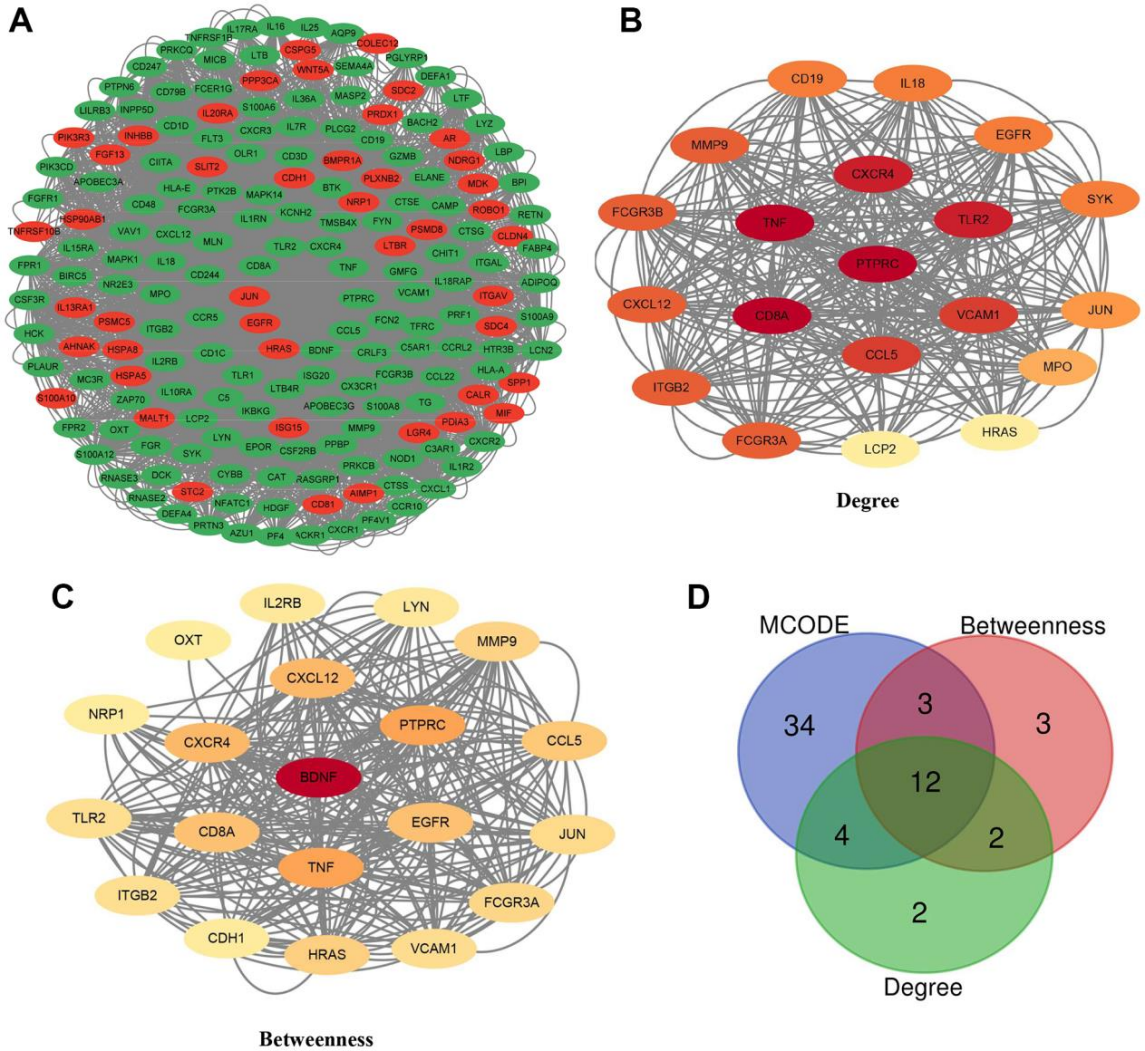
**Hub genes and the biology network for differentially expressed IRGs in GSE32269.** (**A**) A total of 197 IRGs were used for drawing the PPI network. Red indicated up-regulated, and green indicated down-regulated IRGs in bone metastasis tissues of prostate cancer. (**B**, **C**) Confirmation of the top 20 IRGs and establishment of the PPI network by degree and betweenness topological methods. (**D**) Venn diagram to determine 12 hub IRGs.

The STRING website was used to import 209 differentially expressed IRGs, which were then redrawn and optimized using the Cytoscape software (version 3.9.1, java 11.0.6). Genes were ordered by betweenness centrality using the CytoNCA plugin (Figure 3A). There are three key modules, which are degree, betweenness, and MCODE. The top 20 genes selected by the betweenness or degree method and their corresponding networks were mapped (Figure 3B, 3C). Twelve genes were determined as hub genes by taking the intersection of the three methods, which were: FCGR3A, CD8A, CXCR4, VCAM1, HRAS, CCL5, MMP9, CXCL12, ITGB2, PTPRC, TLR2, and TNF (Figure 3D).

### Establishment and validation of a prognostic model

**Figure 4:**
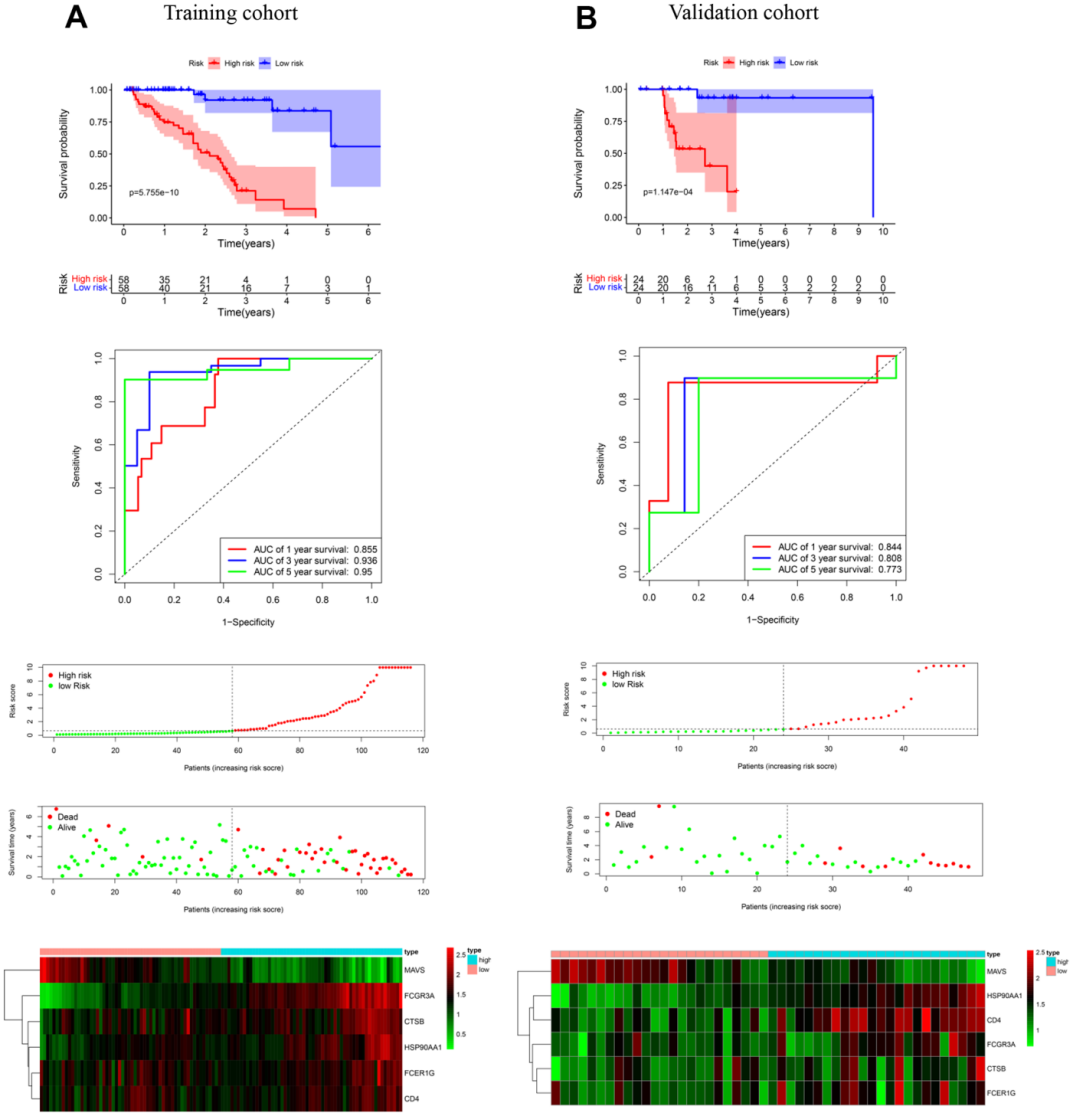
**Establishment and validation of an immune-related gene prediction model for the OS of prostate cancer bone metastases.** (**A**) K-M, ROC, and risk factor analysis were performed to access the association among risk score, mortality, and characteristic gene expression in the training cohort. (**B**) K-M, ROC, and risk factor analysis were performed to access the association among risk score, mortality, and characteristic gene expression in the validation cohort.

For investigating the effect of IRGs on the prognosis of PCa patients with bone metastases, univariate Cox regression analysis was implemented to determine the link between IRGs and OS in the training cohort. 15 OS-related IRGs were filtered out when the standard was set at P < 0.0001 (Figure 1). We selected the top 6 IRGs for further multivariate COX regression analysis. Finally, MAVS, HSP90AA1, FCGR3A, CTSB, FCER1G, and CD4 were obtained for the characters of the OS prediction model. Definition of the model as follows: risk score = (-3.123*exp (MAVS)) + (5.341 *exp (HSP90AA1)) + (6.283 *exp (FCGR3A)) + (4.356* exp (CTSB)) + (3.124*exp (FCER1G)) + (4.252 * exp (CD4)). Each sample in the training cohort was divided into a low- or high-risk group according to the median risk score. Survival analysis illustrated that patients assigned to the high-risk group had a poorer OS than patients assigned to the low-risk group (*p* < 0.001, Figure 4A). To further verify the validity of these prognosis-related genes, a time-dependent ROC analysis was conducted. The areas under the curve (AUCs) at 1, 3, and 5 years were 0.855, 0.936, and 0.95, respectively (Figure 4A). The Survival status diagram and expression heat map were exhibited in Figure 4A.

Importantly, the OS model was applied to the validation cohort from the 30% integrated dataset to validate this. In the validation cohort, the OS was worse in the high-risk group than in the low-risk group (Figure 4B). The AUCs were 0.844, 0.808, and 0.773 for 1, 3, and 5 years (Figure 4B). Finally, the survival status of low- and high-risk patients and expressions of character genes are shown in Figure 4B. In sum, six OS-related IRGs were discerned, and the model for predicting the prognosis of PCa patients with bone metastases was credible.

### Construction and assessment of nomogram for clinical prediction

**Figure 5:**
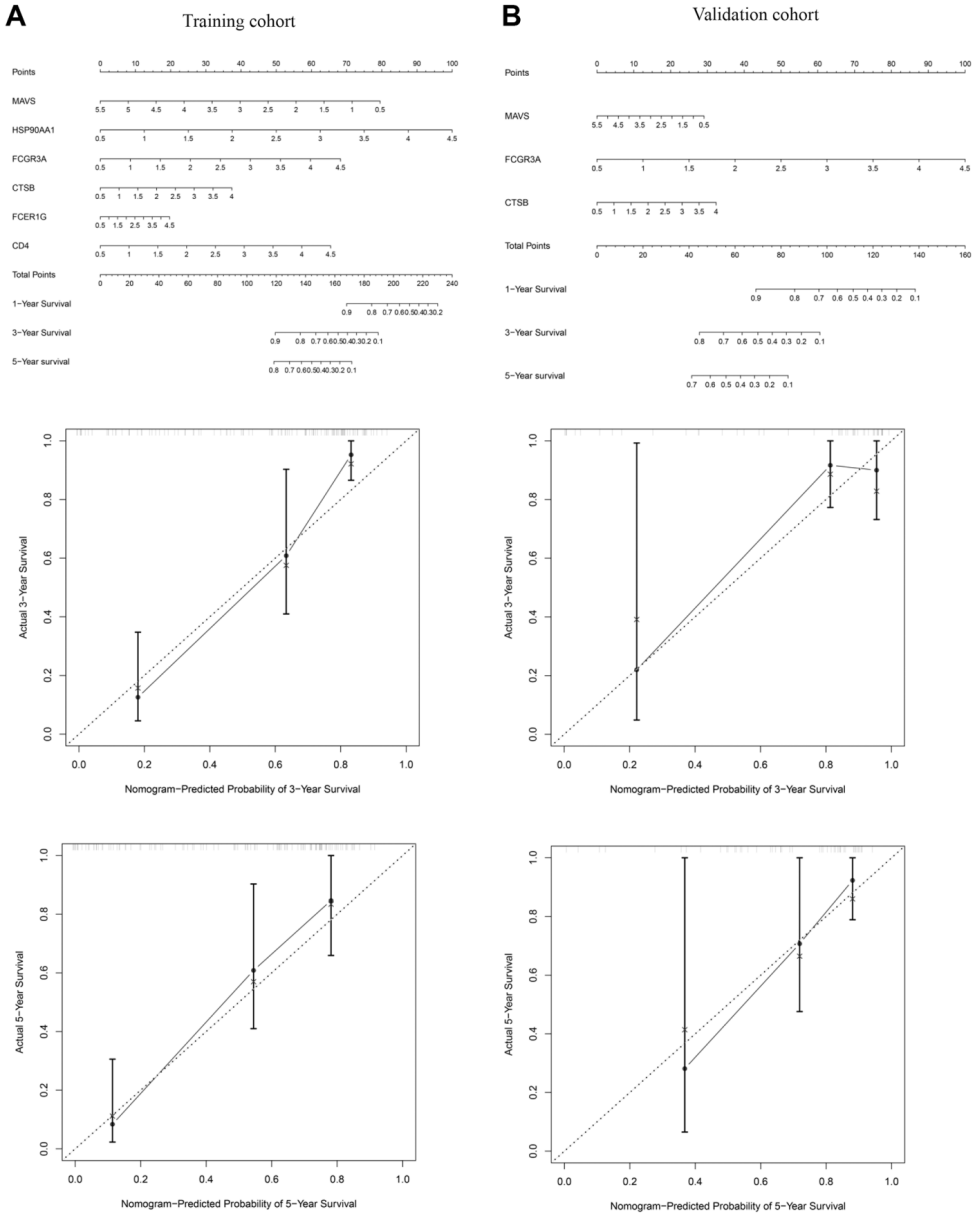
**The nomogram of the OS predictive model for 1, 3, and 5 years.** Nomogram and calibration plot for predicting 1, 3, and 5 years OS model in the training (**A**) and validation (**B**) cohorts.

Nomograms were mapped to establish a practical model that would assist therapists in predicting the OS of PCa-related bone metastases. In the training cohort, six IRGs were integrated, and the nomogram was built to predict patients’ 1-, 3-, and 5-year OS (Figure 5A). A calibration plot was shown to evaluate the accuracy of the predictive model (Figure 5A). Meanwhile, the nomograms and calibration plots for predicting survival rate in the validation cohort were also exhibited in Figure 5B. As we can see from the result, MAVS and FCGR3A play roles in predicting the outcome of patients in the training and validation cohorts (Figure 5A, 5B).

### Tumor-infiltrating immune cells analysis based on the CIBERSORT algorithm

**Figure 6:**
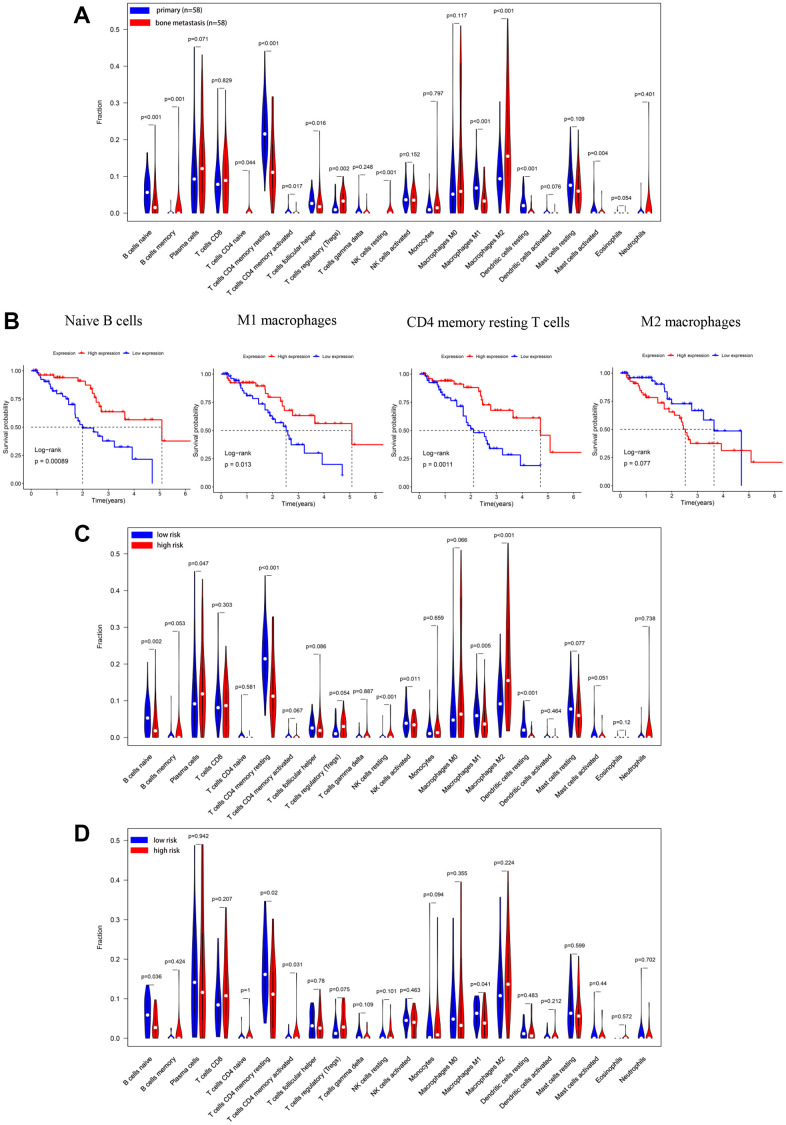
**Immune cells infiltration analysis based on CIBERSORT in PCa bone metastases.** (**A**) The percentage of 22 immune infiltration cells in the training cohort was compared between PCa *in situ* (n =58) and PCa bone metastases (n=58). (**B**) The OS analysis of naive B cells, CD4 memory resting T cells, and M1, M2 macrophages in the training cohort. In the training cohort (**C**) and validation cohort (**D**), the percentages of 22 immune infiltration cells divided into low- or high-risk groups designated by the OS predicted model were compared.

For exploring the status of TIICs in bone metastases of PCa and the influence of risk scores on TIICs, the GSE32269 and GSE77930 datasets were conducted to calculate the proportion of 22 types of TIICs in metastatic bone tissues of PCa by CIBERSORT. Training and validation cohorts were also adopted to investigate the correlation between risk scores and TIICs using this method. For metastatic bone tissues of PCa, M0 and M2 macrophages and plasma cells ruled supreme (Supplementary Figure 1) in the training set. This result was further confirmed in the GSE32269 and GSE77930 datasets (Supplementary Figure 2). Regulatory T cells (Tregs) (*p=*0.002) and M2 macrophages (*p*<0.001) were significantly increased. In contrast, naïve B cells (*p*<0.001), CD4 memory resting T cells (*p*<0.001) and M1 macrophages (*p*<0.001) were significantly decreased in metastatic bone samples of PCa compared to patients with primary PCa (Figure 6A). Further analysis was conducted on the correlation between OS and TIICs. The results indicated that patients with higher naïve B cells (*p=*0.00089, Figure 6B), M1 macrophages (*p=*0.013, Figure 6B), and CD4 memory resting T cells (*p=*0.011, Figure 6B) infiltration ratios had better OS than patients with a lower infiltration ratio. However, patients with a higher M2 macrophage infiltration ratio had worse OS when compared with those with a lower infiltration level, but there was no statistical difference (*p*=0.077, Figure 6B). In the training cohort, compared with the low-risk group, the infiltration ratios of plasma cells (*p*=0.077, Figure 6C) and M2 macrophages (p<0.001, Figure 6C) were significantly increased. In contrast, naïve B cells (*p*=0.002, Figure 6C), CD4 memory resting T cells (*p*<0.001, Figure 6C), activated NK cells (*p*=0.011, Figure 6C) and M1 macrophages (*p*=0.011, Figure 6C) were significantly reduced. We also compared the ratio of immune cell infiltration between low- and high-risk groups in the validation cohort. The results reflected that naïve B cells (*p*=0.036, Figure 6D), CD4 memory resting T cells (*p*=0.02, Figure 6D), and M1 macrophages (*p*=0.041, Figure 6D) were significantly decreased in the high-risk group when compared with the low-risk group. However, the role that these TIICs play in bone metastases of PCa still needs to be further explored.

### Potential immunotherapy targets of prostate cancer bone metastasis

**Figure 7:**
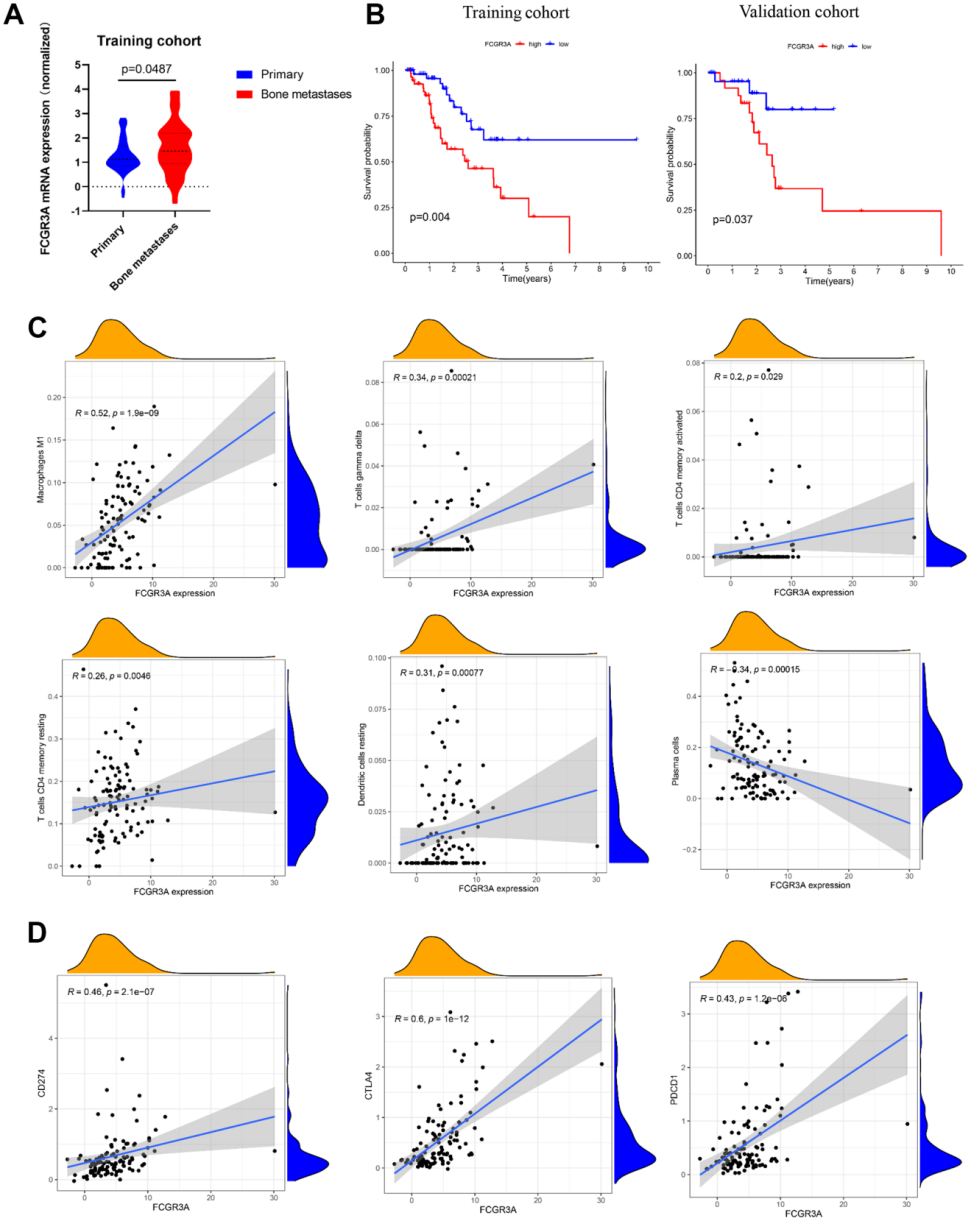
**The linkage between FCGR3A and TIICs.** (**A**) Relative FCGR3A expression level in the normal bone marrow and PCa bone metastases samples in the training set. (**B**) The OS analysis of FCGR3A expression in the training and validation sets. (**C**) Correlation between FCGR3A expression and infiltration of M1 macrophages, gamma delta T cells, CD4 memory activated T cells, CD4 memory resting T cells, resting dendritic cells, and plasma cells in the training set. (**D**) Correlation analysis between FCGR3A expression and immune checkpoints of CD274 (PD-L1), PDCD1 (PD-1), and CTLA4 in the training set.

FCGR3A was screened as a hub gene and prognosis-related gene; therefore, it was investigated further. The FCGR3A expression in PCa bone metastatic tissues was higher than that in the primary PCa tissues (p=0.0487, Figure 7A), and higher FCGR3A was unfavorable to the prognosis of PCa patients in the training set (p=0.004, Figure 7B) and the validation set (p=0.037, Figure 7B). To further investigate the correlation between FCGR3A and TIICs, correlation analysis was conducted in the training set. Results exhibited that FCGR3A expression was remarkably connected with some TIICs (Supplementary Figure 3), such as M1 macrophages (R=0.52, p<0.001, Figure 7C), gamma delta T cells (R=0.34, p<0.001, Figure 7C), CD4 memory activated T cells (R=0.2, p=0.029, Figure 7C), CD4 memory resting T cells (R=0.26, p=0.0046, Figure 7C), resting dendritic cells (R=0.31, p<0.001, Figure 7C) and plasma cells (R=-0.34, p<0.001, Figure 7C). PD-1 (PDCD1) and CTLA4 are two major immune checkpoints on T cells, and they exert their block effect via interacting with PD-L1 (CD274) ligand on PCa cells and CD80/CD86 on antigen-present cells, respectively [31]. Therefore, correlations between FCGR3A expression and PD-1, PD-L1, and CTLA4 expression were analyzed in the training cohort and the TIMER2.0 database (Figure 7D and Supplementary Figure 4). The results reflected FCGR3A expression was positively correlated with PD-L1 (CD274) (R=0.46, p<0.001, Figure 7D), CTLA4 (R=0.6, p<0.001, Figure 7D) and PD-1 (PDCD1/CD279) (R=0.43, p<0.001, Figure 7D) in the training set. We also validated these results in the TIMER 2.0 database. The results were nearly consistent with those in the training set: FCGR3A was positively connected to PD-L1 (CD274) (R=0.577, p<0.001), PD-1 (PDCD1/CD279) (R=0.454, p<0.001) and CTLA4 (R=0.517, p<0.001) (Supplementary Figure 4). All these results exhibited the close relationship between FCGR3A and TIICs; further studies are still urgently needed to investigate the immunologic efficacy of FCGR3A in advanced PCa.

### Up-regulation of MAVS suppressed the proliferation and metastasis of PCa cells

**Figure 8:**
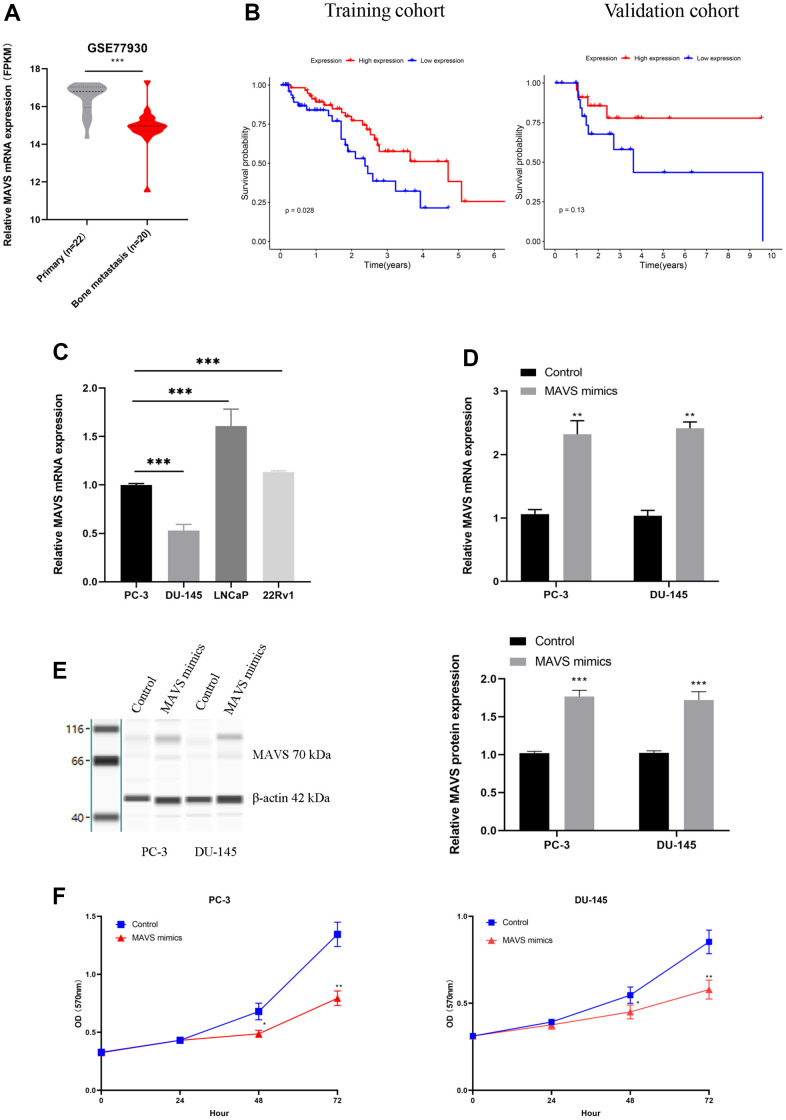
**Up-regulation of MAVS suppressed PCa cells’ proliferation and metastasis.** (**A**) MAVS expression of primary (n=22) and bone metastasis (n=20) of PCa in GSE77930. (**B**) The OS analysis of MAVS expression in the training and validation cohorts. (**C**) Relative MAVS mRNA expression in the PCa cell line. (**D**, **E**) Overexpression efficiency of MAVS in PC-3 and DU-145 cells by RT-qPCR and capillary immunoblotting. (**F**) Assessment of proliferation ability in PC-3 and DU-145 cells with MAVS (controls and mimics) via MTT assay. The original blots are provided in Supplementary Figure 5. *P <0.05, **P <0.01, *** P < 0.001.

Mitochondrial antiviral-signaling protein (MAVS) has been shown to be associated with OS and a protective factor in patients with bone metastases of PCa. However, its role in PCa is currently obscure. As a result, we validated the effect of MAVS on PCa cell lines. MAVS mRNA expression was much lower in bone metastatic tissues when compared to tissues of primary PCa (p<0.001, Figure 8A). Patients with high MAVS mRNA expression had better OS than those with low expression in the training cohort (p=0.028, Figure 8B), but there was no significant statistical difference in the validation cohort (p=0.13, Figure 8B). MAVS mRNA, followed by detection by RT-qPCR in PCa cell lines. MAVS mRNA expression was relatively low in PC-3 and DU-145 cells compared to the other types of PCa cells (Figure 8C). Following this, MAVS mimics and controls were transfected into PC-3 and DU-145 cells. RT-qPCR and Western blotting indicated MAVS expression was significantly increased in PCa cells (PC-3 and DU-145) transfected with MAVS mimics compared with those transfected with control mimics (Figure 8D, 8E and Supplementary Figure 5). MTT assay showed that the proliferation of PC-3 and DU-145 with MAVS mimics was inhibited when compared with the control group (Figure 8F).

**Figure 9:**
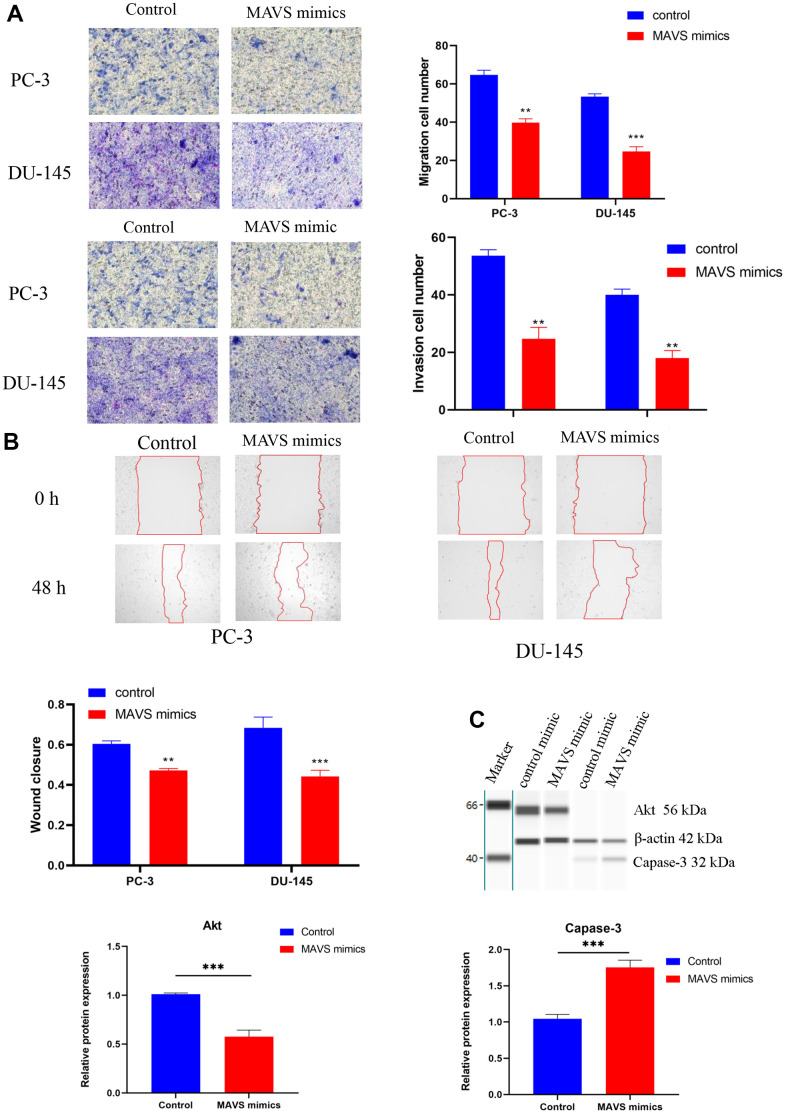
**Up-regulation of MAVS suppressed PCa cells’ proliferation and metastasis.** (**A**) Evaluation of migration and invasion abilities in PC-3 and DU-145 cells with MAVS (control and mimics) via Transwell assay. (**B**) Confirmation of the inhibitory effect of MAVS mimics on PC-3 and DU-145 cells via wound healing assay. (**C**) Capillary immunoblotting analysis of Akt and Capase-3 in PC-3 cells with MAVS (controls and mimics). The original full blots are provided in Supplementary Figure 5. *P <0.05, **P <0.01, *** P < 0.001.

The effect of up-regulated MAVS on PC-3 and DU-145 cell metastasis was also investigated by Transwell and wound healing assays. Results showed that the number of migration and invasion cells with MAVS mimics was markedly decreased in the Transwell assay (Figure 9A). Wound healing assay confirmed that MAVS overexpression could suppress the migration ability of PC-3 and DU-145 cells significantly (Figure 9B). Furthermore, Western blotting showed that up-regulated MAVS could prominently inhibit Akt and increase Capase-3 (Figure 9C and Supplementary Figure 5).

## DISCUSSION

Immunotherapy has employed multiple methods to manipulate or activate natural human immunity, involving the transfusion of specific monoclonal antibodies or immune cells as well as the use of cancer vaccines and cytokines, with the aim of eliminating tumor cells [15]. To date, tumor immunotherapies have achieved great promise in various tumors, offering new and effective choices for patients [15]. Despite the encouraging therapeutic effects of immune checkpoint inhibitors against PD-1, PD-L1, and CTLA-4 across multiple tumor types, the prognosis of metastatic PCa remains unsatisfactory mainly because of drug resistance [32]. Meanwhile, bone metastases are the most significant complication among advanced PCa patients, are ineligible for immunotherapy. Accordingly, further exploration into the role of specific molecular functions and TIICs in PCa bone metastases may suggest new therapy directions for advanced PCa.

In this study, we conducted an integrated analysis of differentially expressed and prognosis-related IRGs in PCa bone metastasis. First, a total of 209 differentially expressed IRGs were filtrated from 2483 IRGs in GSE32269, which concluded that 50 were up-regulated and 159 were down-regulated. We used GO and KEGG enrichment analyses to investigate the function of these IRGs in PCa bone metastases. It turned out that the down-regulated IRGs affected cytokine-cytokine receptor interaction and NK cell-mediated cytotoxicity via cytokine and receptor ligand binding, while the up-regulated IRGs mainly involved T cell leukemia virus infection, antigen processing and presentation, PD-L1 expression, and the PD-1 checkpoint pathway through receptor ligand and cytokine binding. In general, down- or up-regulated IRGs may function differentially in bone metastases of PCa, and they both engage in receptor ligand and cytokine binding. Receptor ligand and cytokine binding are essential processes in the cancer immune response, including PCa [33]. However, the molecular mechanisms underlying PCa bone metastases still need to be explored.

Cytoscape was then used to screen twelve hub genes: FCGR3A, CD8A, CXCR4, VCAM1, HRAS, CCL5, MMP9, CXCL12, ITGB2, PTPRC (CD45), TLR2, and TNF. CXCR4, VCAM1, MMP9, and CXCL12 are involved and play an important role in the peripheral blood NK cells of PCa patients [34]. Significantly, VCAM1 expression was elevated in vascular endothelial cells under the stimulation of IL-17 and insulin/IGF1, which strengthened the adhesion between PCa cells and vascular endothelial cells and promoted prostate cancer metastasis [35]. CCL5 [36], VCAM1, and TLR2 [37] have been demonstrated to be associated with the tumor immune microenvironment and promote PCa cell metastasis. CD8A [38], FCGR3A [39], and PTPRC [40] are identified as candidate biomarkers in various cancers or important molecules in PCa patients with bone metastases. Recent studies have indicated that HRAS alterations in patients with PCa lymph node metastasis demonstrated worse overall survival and disease-free survival [41]. Further studies are still needed to determine how these hub genes contribute to PCa bone metastases.

For studying the effect of differentially expressed IRGs on prognosis, Cox regression analysis was implemented, and an OS model was constructed using the training cohort. First, MAVS, HSP90AA1, FCGR3A, CTSB, FCER1G, and CD4 were selected as characters of the OS prediction model. Then ROC analysis verified that the OS model was reliable when grouping the patients with PCa bone metastases. After that, the OS model was further verified by the validation cohort. Additionally, OS-prediction nomograms were established to make it easier for clinicians to forecast patients’ 1-, 3-, and 5-year survival rates. These results point to the clinical application of the OS prediction model for PCa patients with bone metastases.

Recent studies have demonstrated a strong link between IRGs and TIICs in several tumor types, notably osteosarcoma [16], breast cancer [42], and ovarian cancer [43]. Thus, we looked deeper into the proportion of TIICs and how the risk score affected TIICs in PCa metastatic bone tissues. Results reflected that M0 and M2 macrophages were the main TIICs in metastatic bone tissues of PCa, and M2 macrophages were markedly higher in metastatic bone tissues than in PCa *in situ*. This point can be verified by specific states in which macrophages appear in PCa bone metastases [44]. Additionally, M1 macrophages, naïve B cells, and CD4 memory resting T cells were positively related to OS, which inspired us to activate these TIICs and may contribute to a better prognosis. Some TIICs also correlated with the risk score designated by the OS predictive model, which indicated that these IRGs had an influence on the proportion of TIICs. Even so, further exploration is still needed for these prognosis-related IRGs.

FCGRs constitute the receptor for the Fc segment of immunoglobulin, which is composed of three important parts: FCGR I, FCGR II, and FCGR III. The genes encoding FCGRs are highly polymorphic and involved in various biological processes, including aggregating immunoglobulin, phagocytosis, and antibody-dependent cellular cytotoxicity [45, 46]. FCGR3A is a crucial component of the FCGRs family, and it is restricted to being expressed in natural killer (NK) cells and monocytes/macrophages [45]. It encodes a transmembrane receptor that allows the immune cells to recognize and kill targeted cells [47]. Recently, researchers have found that FCGR3A is highly expressed in pan-cancer, including PCa, and it could be an independent biomarker for PCa patients [39, 48]. In this study, FCGR3A was identified as a hub gene and prognosis-related gene via PPI and Cox regression analyses. Further, we found that, when compared to the PCa primary tissues, FCGR3A was highly expressed in bone metastatic tissues. Interestingly, results suggest FCGR3A was markedly positively correlated with M1 macrophages and T cells but not with NK cells and monocytes in PCa bone metastatic tissues, which may indicate that NK cells and monocytes were not dominant and FCGR3A may be mainly expressed in macrophages in bone metastases of PCa. It is also vital to note that FCGR3A positively correlates with other biomarkers and is a key target for medications like rituximab [49]. This study discovered a substantial relationship between FCGR3A and immune checkpoints, including PD-1, PD-L1, and CTLA4, suggesting FCGR3A would be a promising immunotherapy target for patients with advanced PCa.

While developing an OS predictive model for patients with bone metastasis, we observed that MAVS acted as a prognostic protective factor while other IRGs acted as prognostic risk factors. The function of MAVS in PCa bone metastases remains unknown. Thus, preliminary experiments were conducted to explore the effect of MAVS on PCa cells. Results revealed that MAVS inhibited the proliferation, migration, and invasion of PCa cells. Accordingly, MAVS may play an important role in PCa progression and can be a practical biomarker for predicting the prognosis of PCa patients with bone metastases. Previous studies have reported that MAVS can be activated by exogenous virus RNA and exert its anti-tumor effect by up-regulating the downstream pro-apoptotic genes TRAIL and Noxa to induce apoptosis in PCa cells [50, 51]. To verify this, we also detected the expression level of apoptosis-related proteins. The results showed that up-regulated MAVS could also promote the expression of apoptosis-related protein Capase-3 in PC-3 cells. Previous studies have illustrated that the PI3K/AKT pathway is completely deregulated in advanced PCa [52], and this pathway also serves as a key player in the anti-apoptotic role [53]. We hypothesized that MAVS could exert its role in the PI3K/AKT pathway in PCa cells. The finding suggests that activation of MAVS could suppress the expression of Akt in PC-3 cells.

Although we used bioinformatics analysis to identify hub IRGs and constructed a prognostic prediction model, this study is subject to some limitations. First, more experimental research should be implemented at the cellular level, such as using flow cytometry to explore the role of MAVS in the cell cycle and apoptosis of PCa. Second, animal models are indispensable for researching the etiology of cancer bone metastases and facilitating effective treatment strategies [54]. Currently, the most commonly used *in vivo* models for studying the bone metastasis of PCa include animal models, cell line injection models, and bone-implant models [55], while cell line injection models are the most commonly used *in vivo* models [55]. Therefore, in a follow-up experiment, cell line injection models will be adopted to explore the underlying mechanisms of MAVS for PCa bone metastasis *in vivo*. Last but not least, limited to the number of samples from patients with PCa bone metastasis in the cBioPortal database, this study only enrolled 83 patients with bone metastasis; thus, more datasets with clinical prognostic information need to be analyzed to make the prediction model more accurate.

In summary, we conducted a comprehensive study of the role of hub genes in bone metastasis of PCa and their potential immunotherapy values. The OS prediction model that can accurately predict the OS of PCa patients with bone metastasis was established by the training cohort and verified via the validation set. Six OS-related IRGs and twelve hub genes were recognized. FCGR3A and MAVS could be effective therapeutic targets in the bone metastasis of PCa.

## Supplementary Material

Supplementary Figures

Supplementary Tables 1 and 2

Supplementary Table 3

Supplementary Table 4
