# Supplementary material for: Identification and validation of a novel overall survival prediction model for immune-related genes in bone metastases of prostate cancer
Source: Aging (Albany NY). 2023 Jul 25;15(14):7161–86. doi: 10.18632/aging.204900 (PMC10415549; doi:10.18632/aging.204900)
Supplement: Supplementary Figures [file aging-15-204900-s001.pdf]

SUPPLEMENTARY FIGURES

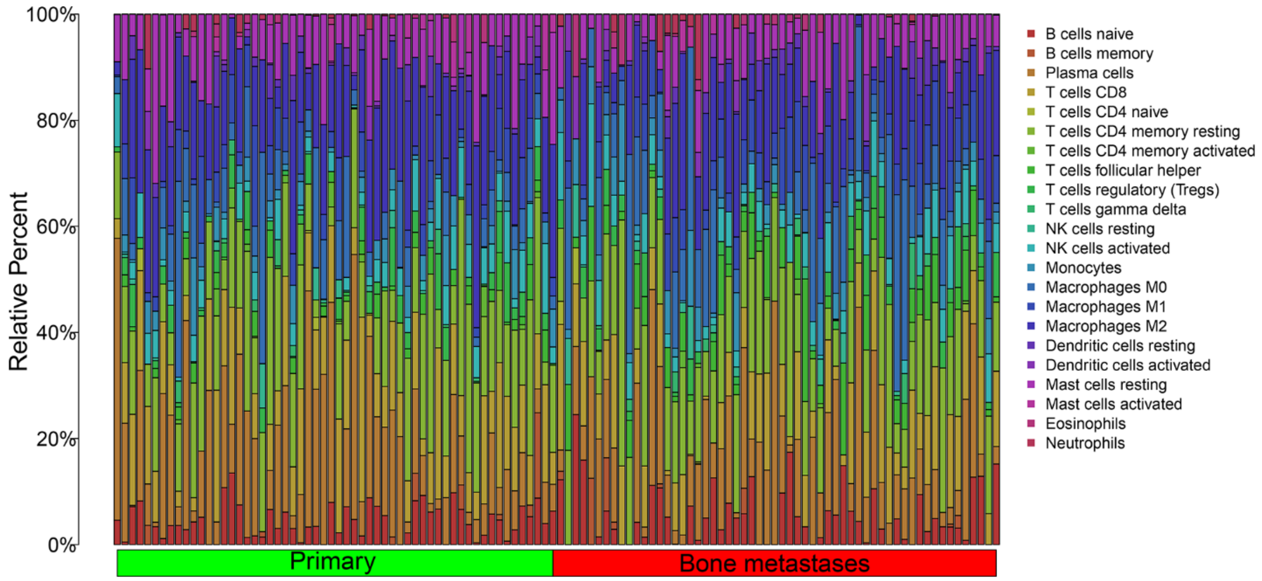

Supplementary Figure 1. The proportion of 22 types of TIICs was compared between primary and bone metastases of PCa in the training set.

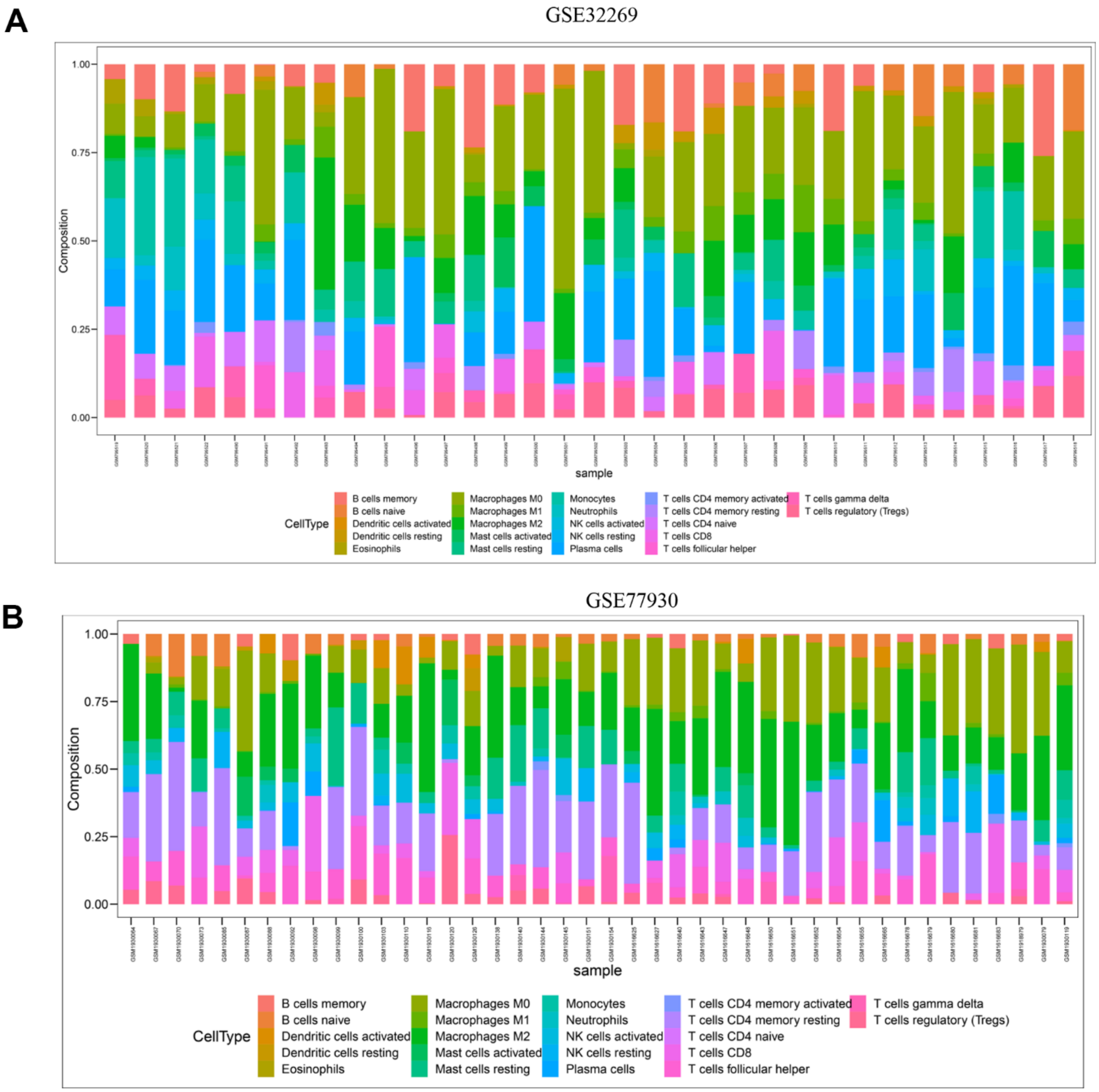

Supplementary Figure 2. The proportions of 22 TIICs in each sample of GSE32269 (**A**) and GSE77930 (**B**) are exhibited.

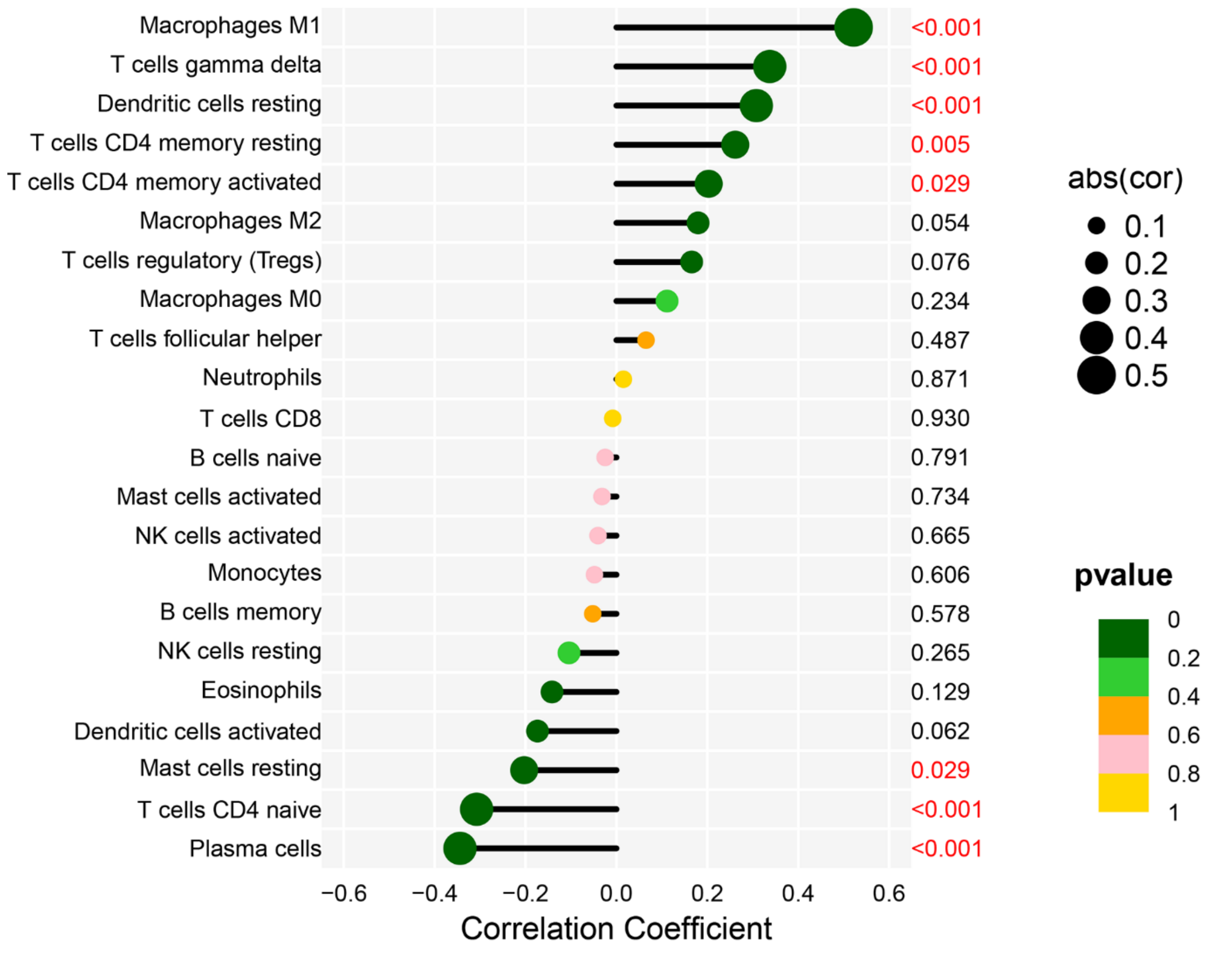

Supplementary Figure 3. Correlation analysis between FCGR3A expression and 22 types of TIICs in the training set.

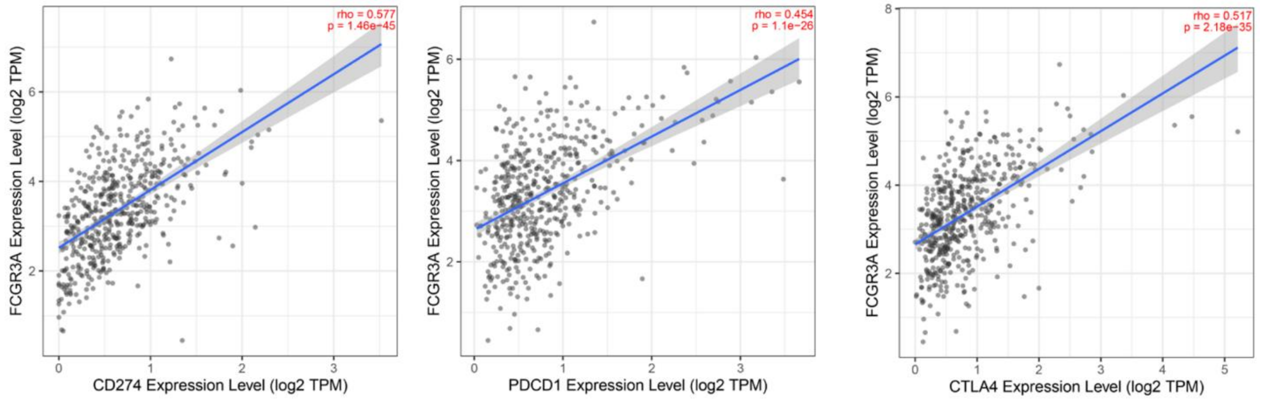

Supplementary Figure 4. In the TIMER2.0 database, correlations were analyzed between FCGR3A expression and CD274 (PD-L1), PDCD1 (PD-1), and CTLA4 expression, respectively.

Figure 7E original WB image

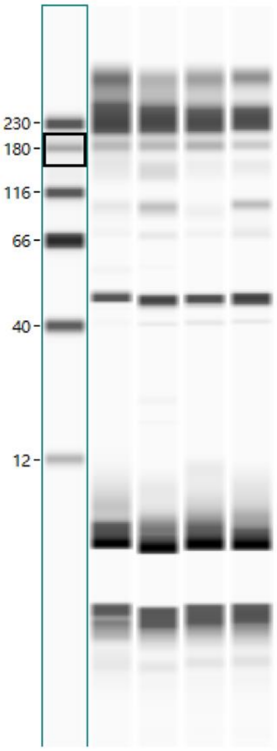

Cutting position

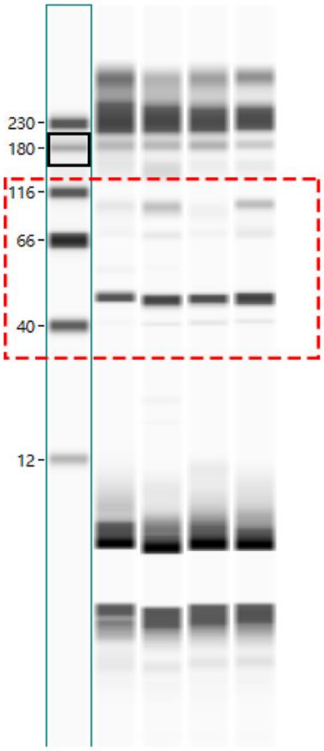

Figure 7E

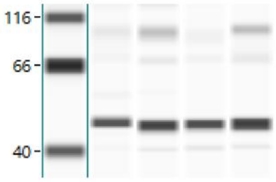

Figure 8C original WB image

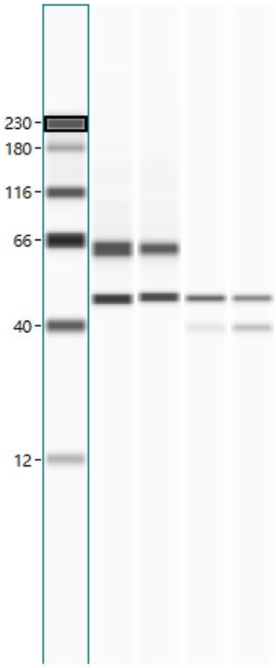

Cutting position

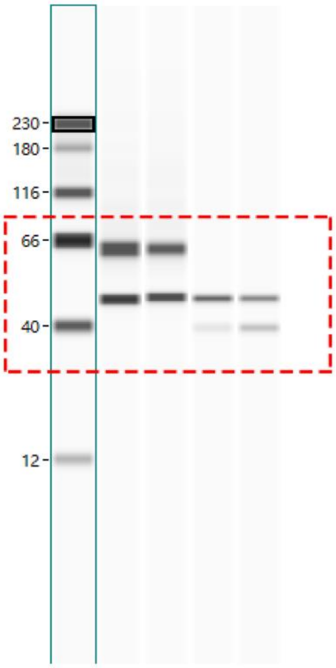

Figure 8C

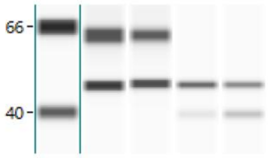

Supplementary Figure 5. The full images of the original immunoblots.
