# Supplementary material for: Identification and validation of a novel overall survival prediction model for immune-related genes in bone metastases of prostate cancer
Source: Aging (Albany NY). 2023 Jul 25;15(14):7161–86. doi: 10.18632/aging.204900 (PMC10415549; doi:10.18632/aging.204900)
Supplement: Supplementary Tables 1 and 2 [file aging-15-204900-s002.pdf]

SUPPLEMENTARY TABLES

Supplementary Table 1. Primer sequences.

Gene	Primer sequences (5'-3')
MAVS	Forward: CAGGCCGAGCCTATCATCTG<br>Reverse: GGGCTTTGAGCTAGTTGGCA
GAPDH	Forward: CACCATCTTCCAGGAGCGAG<br>Reverse: TCACGCCACAGTTTCCCGGA

Supplementary Table 2. 209 differentially expressed IRGs in GSE32269.

50 up-regulated IRGs in bone metastasis of prostate cancer.

CALR	PDIA3	HSPA5	HSPA8	HSP90AB1	PSMC5	PSMD8	TMSB15A	S100A10	LMBR1L
COLEC12	ISG15	JUN	ITGAV	CD81	WNT5A	AHNAK	TNFRSF10B	CLDN4	NDRG1
PRDX1	CDH1	MIF	PPP3CA	HRAS	MALT1	PIK3R3	SLIT2	PLXNB2	ROBO1
CSPG5	FAM3C	FGF13	INHBB	MDK	AIMP1	SPP1	STC2	ACVRL1	SDC2
AR	BMPR1A	EGFR	IL13RA1	IL20RA	LGR4	LTBR	NR2F6	NRP1	SDC4

159 down-regulated IRGs in bone metastasis of prostate cancer.

CD1C	CD1D	CD8A	CTSE	CTSS	FCER1G	HLA-A	HLA-DRB4	HLA-E	CIITA
MICB	CAMP	PPBP	CXCL1	CXCL12	PF4	ELANE	DEFA1	DEFA4	LCN2
BPI	S100A9	S100A8	S100A12	CCR10	TMSB4X	PGLYRP1	S100A6	S100P	AZU1
TINAGL1	PF4V1	MMP9	APOBEC3G	TLR2	LBP	LTF	FABP4	TNF	CTSG
PRTN3	MAPK1	CYBB	ISG20	TFRC	ADIPOQ	CHIT1	FCN2	LYZ	CCL5
MAPK14	NOD1	TLR1	MPO	DCK	KCNH2	RNASE3	IL18	LTB4R	APOBEC3A
MASP2	IL7R	BACH2	AQP9	BIRC5	VCAM1	CXCR1	CCL22	CCR5	CXCR4
PTK2B	FGR	HCK	OLR1	RNASE2	CD79B	LYN	SYK	BTK	VAV1
NFATC	IKBKG	CD19	PIK3CD	INPP5D	PTPN6	LILRB3	PLCG2	PRKCB	IGHA1
IGHD	IGHG1	IGHM	IGKC	IGKV1-17	IGLC1	IGLJ3	IGLV1-44	C5	SEMA4A
C5AR1	CCRL2	CX3CR1	CXCR3	ACKR1	FPR1	FPR2	CXCR2	PLAUR	BDNF
CAT	CGB1	GMFG	HDGF	IL16	IL36A	IL1RN	IL25	LTB	MLN
OXT	RETN	TG	C3AR1	CRLF3	CSF2RB	CSF3R	EPOR	FGFR1	FLT3
HTR3B	IL10RA	IL15RA	IL2RB	IL17RA	IL18RAP	IL1R2	MC3R	NR2E3	PTGER2
TNFRSF1B	ITGAL	ITGB2	FCGR3A	FCGR3B	CD247	ZAP70	LCP2	FYN	CD48
CD244	GZMB	PRF1	CD3D	PTPRC	RASGRP1	PRKCQ	TRAC	TRBC1	
